# Opportunities and Challenges of Integrating Ethiopian Traditional Medicine System Into Modern Medicine: A Narrative Review

**DOI:** 10.1155/tswj/8106682

**Published:** 2026-07-01

**Authors:** Kassahun Dires Ayenew, Berihun Alem, Birhan Mengstie, Awgichew Shewasinad Yehualashet

**Affiliations:** ^1^ Department of Pharmacy, Asrat Woldeyes Health Science Campus, Debre Berhan University, Debre Berhan, Ethiopia, dbu.edu.et

**Keywords:** challenges, Ethiopia, integration, knowledge preservation, opportunities, regulation, traditional herbal medicine

## Abstract

**Background:**

About 80% of the population uses Ethiopian traditional herbal medicine (ETHM) for a variety of medical needs, making it a pillar of the country′s healthcare system. The advantages and challenges of integrating ETHM with contemporary medicine in Ethiopia are summed up in this narrative review.

**Methods:**

To explore the integration of ETHM with contemporary medical systems, we carried out a literature search in March 2025 while maintaining methodological transparency. Using a mix of keywords associated with ETHM, integration, obstacles, and pharmacological validation, we searched a number of databases, including PubMed, Scopus, African Journals Online (AJOL), and Google Scholar. A total of 110 records were found in the first search. After duplicates were eliminated, and titles and abstracts were evaluated for relevancy, 65 items remained. After a thorough analysis of the remaining literature, we included 38 articles published between January 2015 and March 2025 that satisfied our predetermined inclusion criteria, which were centered on ETHM practices, integration with contemporary medicine, or Ethiopian regulatory frameworks. Studies and publications written in languages other than English that did not particularly discuss ETHM or its integration were excluded. We critically assessed each included study′s relevance and trustworthiness based on elements like journal impact factor, authorship, and methodological rigor, even though our narrative evaluation did not employ a rigorous systematic quality assessment.

**Results:**

A strong basis for integration is identified by our synthesis, which includes a large pharmacopeia of medicinal plants with encouraging preclinical evidence for conditions including diabetes and wound healing. However, progress is hampered by systemic problems. These include significant problems with product quality and adulteration, a dearth of clinical validation and safety data, a lack of a thorough regulatory framework, and a high degree of mistrust between traditional practitioners and contemporary scientific groups.

**Conclusion:**

In order to document and preserve indigenous knowledge, we advise the Ethiopian Food and Drug Administration (EFDA) to immediately establish a dedicated directorate for traditional herbal medicine and to launch a national digital repository called “EthHerb.”

## 1. Introduction

### 1.1. Background

The World Health Organization (WHO) defines traditional medicine as all knowledge, skills, and methods derived from indigenous theories, beliefs, and experiences that are used to maintain health and prevent, diagnose, treat, or improve physical and mental illnesses [1].

Traditional medicine, which has ancient roots and integrates natural and spiritual reasons for health, is used by 80% of Ethiopians [2, 3]. Between 6500 and 7000 species of higher plants are believed to exist in Ethiopia, with 12% of them being native species. Additionally, about 90% of traditional medicinal compositions are derived from plants [4]. The integration of traditional and modern methods is nevertheless hampered by policy gaps, despite an increase in government assistance since the late 20th century. Ethiopian traditional medicine not only cures illness but also safeguards and enhances material, mental, spiritual, social, and physical well‐being. Traditional medicine, which has ancient roots and integrates natural and spiritual reasons for health, is practiced by 80% of Ethiopians [2, 3]. The number of higher plant species in Ethiopia is estimated to be between 6500 and 7000, of which 12% are native. Additionally, about 90% of traditional medicinal compositions come from plants [4]. Despite an increase in government support since the late 20th century, policy gaps continue to impede the integration of modern and ancient practices. Ethiopian traditional medicine not only treats illness but also safeguards and enhances material, mental, spiritual, social, and physical well‐being.

Despite being one of the world′s oldest countries and having a long history of employing herbal medicine, Ethiopia′s application and regulatory framework lacks sufficient proof . According to a study evaluating Ethiopia′s herbal medicine rules, the government has not yet passed particular laws and policies that would provide an autonomous regulatory framework. Even though the Ethiopian Food and Drug Administration (EFDA) has mandated the regulation of herbal medicines, further study is necessary because traditional healers are licensed by woredas and regional health bureaus [3].

Ethiopian traditional healers have historically used indigenous knowledge systems that combine spiritual and ritual aspects of treatment with empirical herbal knowledge (e.g., using aloe for burns, ginger for nausea, and artemisia for malaria). Even if contemporary medicine is gradually being incorporated, the nation′s healthcare system still mainly depends on conventional methods. Research and ancient pharmacopeias show that Ethiopians have employed plants for a variety of therapeutic uses [5]. The opportunities and difficulties of integrating Ethiopian traditional medicine with contemporary medicine were reviewed in this review.

### 1.2. Statement of the Problems

Since 80% of the population uses Ethiopian traditional herbal medicine (ETHM) as their primary form of therapy, it is an essential component of rural healthcare [6]. Because of systemic issues like a lack of scientific validation, quality control, and regulatory monitoring, ETHM is still not fully incorporated into contemporary pharmaceutical systems despite its widespread use [3, 4]. Less than five clinical trials on medicinal plants have been carried out since 2015, and only 12% of them have undergone pharmacological analysis, according to research, compromising their validity in contemporary medicine [3, 7].

Significant safety concerns have also been raised by the discovery that about 30% of herbal products in Addis Ababa include pollutants such as heavy metals, microbiological infections, or unreported synthetic additions [8]. These dangerous substances can come from a variety of sources: (i) environmental contamination (such as heavy metals from soil or water); (ii) adulteration by suppliers or vendors looking to increase efficacy or cut costs; and (iii) inconsistent preparation practices among some practitioners, even though traditional healers possess extensive indigenous knowledge of plant properties. According to regional pharmacovigilance records, hepatotoxicity and renal impairment associated with contaminated herbal products have been documented as patient harm [1]. The commercial and unregulated nature of some herbal markets poses problems beyond their control, even if indigenous healers have extensive knowledge of plant toxicity and safe use, as evidenced by ethnographic studies such as [9] on healer knowledge of plant toxicity.

The loss of indigenous knowledge is a major threat to the maintenance of traditional practices, as 68% of ethnobotanical knowledge is kept by old healers and has accelerated by 42% in the previous 15 years owing to urbanization [10]. Furthermore, only 40% of the goals of the 2019 Traditional Medicine Policy have been achieved, mostly due to uneven state and municipal implementation and a lack of funds [11]. Unsustainable harvesting practices, habitat loss, and environmental degradation pose a threat to two‐thirds of medicinal plants that are gathered from the wild. These include valuable species such as *Echinops kebericho* and *Artemisia annua*, whose yields have decreased by 18% due to climate change [12, 13]. The long‐term availability of the medicinal flora is threatened by these ecological pressures, which further complicate integration efforts.

Surprisingly, 61% of Ethiopians reported using herbal medicine more frequently following COVID‐19, suggesting that demand for this treatment is still growing. Ethiopia accounts for less than 1% of the global market trend, which is expected to reach $178 billion by 2026 due to a lack of strategic integration [1, 14]. Closing the gap in scientific evidence, amending legislation pertaining to traditional medicine, and using digital technologies like EthHerb AI to document and preserve indigenous knowledge are the three main solutions highlighted in this study to encourage systemic improvements.

This narrative review is aimed at exploring the opportunities and problems of integrating Ethiopian traditional medicine with contemporary healthcare. In particular, it is aimed at analyzing current research to pinpoint potential advantages and important knowledge gaps about Ethiopian traditional medicine, assessing the difficulties encountered in integrating it, evaluating the regulatory and policy frameworks that oversee this integration—including problems with standardization and implementation—and, finally, offering evidence‐based suggestions for successfully integrating traditional medicine into modern healthcare systems through improved policies, research projects, and cooperative efforts.

## 2. Methods and Materials

### 2.1. Literature Search and Selection Strategy

In March 2025, we carried out a literature search to give a thorough review of the integration of ETHM with contemporary medical systems. We synthesized existing literature while keeping our technique transparent.

#### 2.1.1. Databases Searched

The following databases were utilized in this review:

• PubMed
• Scopus
• African Journals Online (AJOL)
• Google Scholar

#### 2.1.2. Search Dates

The search was conducted in March 2025.

#### 2.1.3. Search Strings

Several keyword combinations were taken into consideration, such as “Ethiopia” AND (“traditional medicine” OR “herbal medicine”). “Ethiopian traditional medicine” AND (“integration” OR “policy” OR “regulation”) “Ethiopia” AND (“challenges” OR “opportunities”) “pharmacological validation.”

#### 2.1.4. Number of Articles Retrieved

A total of 110 entries were found in our first search.

#### 2.1.5. Screening Process

Sixty‐five articles remained at this point after duplicates were eliminated, and titles and abstracts were evaluated for applicability.

#### 2.1.6. Final Included Studies

After carefully examining the remaining papers, we selected 38 researches that satisfied our inclusion requirements.

### 2.2. Inclusion/Exclusion Criteria

#### 2.2.1. Inclusion Criteria

• Research on Ethiopian regulatory frameworks, ETHM practices, or integration with contemporary medicine.
• Articles published between January 2015 and March 2025.

#### 2.2.2. Exclusion Criteria Included

• Publications not available in English
• Articles that did not specifically address ETHM or its integration with modern medical systems

### 2.3. Quality Assessment

We critically assessed the applicability and reliability of each included study based on the journal′s impact factor, authorship, and methodological rigor, even though this narrative review does not use a rigorous systematic quality evaluation.

## 3. Results

### 3.1. Overview of the Current Evidence Base

This narrative review summarizes research on ETHM′s integration with contemporary healthcare from 2015 to 2025. The evidence base is broad and includes public health research, preclinical investigations, ethnobotanical surveys, and policy assessments. The important discoveries are summarized thematically in the following parts, with a selection of papers demonstrating the depth of existing knowledge.

### 3.2. Ethiopian Traditional Medicine Practices and Self‐Medication

Recent polls show that almost 80% of Ethiopians have used herbal remedies for basic medical requirements at some point, with a sizable percentage being active or frequent consumers. This illustrates how traditional medicine is still crucial to the nation′s healthcare system [15]. The widespread use of therapeutic plants like *Moringa stenopetala* for diabetes and *Allium sativum* for hypertension demonstrates the ongoing relevance of ethnomedical knowledge [16]. Despite ongoing worries about safety and standardization, traditional healers continue to play a significant role in providing healthcare in rural regions by using both herbal remedies and spiritual practices [17]. The government′s 2013 Traditional Medicine Policy has been slowly implemented; current efforts focus on integrating effective traditional medicines into primary care settings [18].

There are extra hazards associated with herbal self‐medication because 12% of traditional remedies examined in Addis Ababa markets had heavy metal contamination, according to recent toxicological assessments [19]. Experts stress the necessity for public education campaigns and more stringent regulation of both pharmaceutical and herbal goods, even if cost and accessibility are the main factors driving self‐medication behaviors [20].

According to recent research, if appropriate quality controls are put in place, including traditional medicine within the official healthcare system may help lower the rate of risky self‐medication [21]. Only 15% of traditional healers had official training as of 2021, indicating that there are still issues with standardizing preparations and training practitioners [22]. While addressing safety issues, ongoing clinical investigations of Ethiopian medicinal plants at establishments such as Addis Ababa University may aid in the validation of traditional knowledge [23].

### 3.3. Opportunities of the Integration

#### 3.3.1. For Scientific Validation and Standardization of Herbal Medicines

The standardization and scientific confirmation of Ethiopian traditional remedies present a significant possibility for their integration into contemporary medicine. The quality, safety, and effectiveness of these treatments can be assessed by researchers using exacting, evidence‐based techniques from contemporary pharmacology. Through this approach, traditional knowledge is not only maintained but also given additional scientific legitimacy, which increases its acceptability in modern healthcare systems [24].

Data‐driven approaches such as association rule mining and in silico techniques can be used to analyze traditional medicine practices in order to promote this integration. These technologies help uncover patterns between the herbal components used and commonly treated diseases, thereby creating a reliable database for practitioners. Moreover, molecular‐level research can reveal biological processes, disease mechanisms, and treatment targets. *Rumex abyssinicus* study has demonstrated remarkable wound‐healing properties, providing a notable example of how traditional remedies may be in line with current scientific discoveries [24].

According to 57.8% of respondents, perceived low quality is a significant problem in Ethiopia′s traditional herbal medicine sector. Furthermore, over half of those surveyed expressed dissatisfaction with key aspects of herbal treatments, such as their efficacy (54.0%), safety (51.9%), quality (58.2%), and appropriate use (59.9%). In every significant category, including storage conditions, 49.8% of respondents rated the system poorly. In order to increase the legitimacy and safety of traditional herbal medicine in Ethiopia, these findings emphasize the urgent need for improved regulatory monitoring, standardization, and quality control [4].

#### 3.3.2. For Advanced Drug Discovery and Development Through Integration

In contemporary pharmacology, technological advancements and computational modeling have significantly sped up the drug discovery and development process. These technologies could potentially be applied to accelerate the study of Ethiopian medicinal plants. [25]. ETHMs can benefit from these developments since computational drug discovery, sometimes referred to as in silico drug discovery, uses virtual screening (VS) and ligand‐based drug design (LBDD), both of which use computational methods to speed up the identification and design of medicinal compounds [24].

#### 3.3.3. For Regulatory Framework Development

A study highlights the need for thorough policy development, improved training initiatives, and fortified regulatory frameworks in order to effectively monitor and regulate traditional herbal medicine practices in Ethiopia. It is important to clarify that Ethiopia is not operating in a regulatory vacuum. Although significant gaps remain, the EFDA has undertaken ongoing efforts to develop and strengthen the regulatory framework for herbal medicines, as noted in recent evaluations [4]. However, these initiatives are still in development and face challenges in implementation. By encouraging evidence‐based research and collaboration between scientific communities, regulatory bodies, and traditional healers, plans could enhance Ethiopia′s regulation of traditional herbal medicine. The advancement of the safety and quality of medications derived from herbal treatments depends on these collaborative efforts. Since the current regulatory frameworks for herbal medicine in many African countries are inadequate and underdeveloped, Ethiopia could establish a robust system [4].

#### 3.3.4. For Interdisciplinary Collaboration

The effective incorporation of traditional medicine into modern healthcare requires close interdisciplinary collaboration between scientific groups, regulatory agencies, and traditional healers. Creating a bridge between these parties facilitates mutual understanding and enables the implementation of standardized, effective therapies. Future progress will depend on dismantling long‐standing barriers and encouraging cooperation between basic researchers and physicians, academia and industry, and throughout the healthcare system. Equally important is the growing involvement of patients in pharmaceutical research. Their engagement promotes pharmaceutical development with patient‐reported outcomes and influences research agendas through lobbying. The shift from passive recipients to active participants enhances the feasibility, efficacy, and relevance of pharmacological advancements, ultimately leading to more patient‐centered healthcare solutions [26].

#### 3.3.5. Training and Capacity Building for Practitioners

The lack of state‐recognized or standardized training for regulatory personnel, 82.3% of whom say they have no prior expertise in this area, is a serious flaw in Ethiopia′s regulations governing traditional herbal medicine. This suggests that there is a significant opportunity to boost capacity through targeted training programs. Giving regulatory staff and traditional healers training in areas like pharmacology, herbal formulation, and patient care can increase the effectiveness and safety of conventional therapies.

It is important to clarify that traditional healers in Ethiopia, as in many cultures, undergo extensive apprenticeship‐based training embedded within cultural, spiritual, and community‐based systems of knowledge transmission. This form of training is deeply respected and recognized within their communities. However, the term “official training” in the context of this manuscript refers to state‐sanctioned professionalization and certification programs that could complement, not replace, these traditional pathways. Such programs are aimed at standardizing aspects of safety, quality assurance, and integration with modern healthcare systems, while respecting and preserving indigenous knowledge systems. Furthermore, by receiving supplementary, formalized training, traditional healers can modify their practices to better suit contemporary healthcare systems while maintaining cultural legacy. Aspiring medical professionals are more respectful and understanding when traditional medicine is incorporated into educational programs. This teaching approach promotes a more inclusive and holistic vision of healthcare while acknowledging both recent research and conventional wisdom [4].

#### 3.3.6. For Sustainability and Conservation of Medicinal Plants

Ethiopia′s enormous biodiversity, which comprises 6500–7000 plant species, 10%–12% of which are indigenous, makes traditional medicine a popular practice there. More than 1000 of these species are used medicinally, reflecting the nation′s long‐standing reliance on herbal remedies. This natural abundance offers a great foundation for integrating traditional medicine into modern treatment. However, increased use leads to sustainability problems, particularly in relation to overharvesting of plants and biodiversity loss. Integrating old methods with modern procedures can help to promote environmental stewardship [24].

#### 3.3.7. Conclusion About the Opportunities of the Integration

Modern pharmaceutical systems and ETHM can be combined to enhance healthcare, protect cultural heritage, and advance environmental sustainability. Ethiopia is able to develop a more comprehensive and efficient healthcare plan by fusing scientific advances with traditional wisdom. A multifaceted strategy including quality control, scientific validation, regulatory reform, education, and ethical cooperation will be needed to achieve this integration. With consistent funding and cross‐sector partnerships, this review could inform policies that strengthen Ethiopian healthcare and contribute to global understanding of traditional medicine integration [4].

### 3.4. Challenges of the Integration

#### 3.4.1. Regulatory Framework Challenges

Ethiopia′s current national medicine declaration is said to efficiently govern traditional herbal medicine (ETHM). One of the primary issues is that the same regulatory agency cannot apply the same standards to both conventional and traditional medicines, which creates significant challenges. The lack of clear definitions for ETHM procedures and products, as well as unclear legislation relating to ETHM regulatory requirements, further hinders effective oversight. Furthermore, the absence of a dedicated regulatory body or unit with specialized oversight for ETHM within the EFDA contributes to the inadequate application of ETHM regulation [3].

These regulatory deficiencies are made worse by regional ETHM directives, which primarily focus on regulating practitioners and facilities while neglecting the broader range of ETHM services and herbal treatments created by traditional healers. National efforts to ensure the quality, safety, and efficacy of traditional herbal medicines are put at risk by the uneven application of regulatory standards in various regions. The actual implementation of the EFDA′s draft model directive to direct ETHM regulation has been fraught with difficulties [2]. Furthermore, there is no official oversight of Ethiopia′s traditional herbal veterinary care. Veterinary ETHM practices are unregulated since the Veterinary Drug and Animal Feed Administration and Control Authority has not created or implemented any legislation in this area. This regulatory gap highlights the need for a comprehensive and context‐specific institutional and legal framework to ensure the safe and effective incorporation of traditional herbal medicine for humans and animals into the nation′s healthcare system [3].

#### 3.4.2. Administrative Structure Deficiencies

Oversight is made very challenging by Ethiopia′s disjointed and inconsistent administrative system for regulating traditional herbal medicine (ETHM). The EFDA is in charge of herbal medicine products, whereas the Regional Health Regulatory (RHR) offices oversee ETHM operations and practitioners. It is challenging to establish unified regulation and incorporate ETHM with traditional medicine in this disjointed system since there are no research‐based approval procedures for ETHM procedures and products. Regulatory offices face severe resource restrictions, including inadequate money, specialized staff, and proper facilities for ETHM oversight. Additionally, there is no separate ETHM division at the federal level or a single administrative structure linking federal and regional offices. Because of these structural flaws, different regions have different regulating approaches, which hinders the growth of a strong national ETHM system [4].

Despite these challenges, certain regions, like Oromia, have made headway since the 2007 E.C. implementation of registration and licensing procedures for ETHM practitioners and enterprises. Local, zonal, and regional officials each have a portion of the regulatory responsibilities in these areas. However, without a single national framework and sufficient funding, such regional initiatives continue to be dispersed and insufficient to ensure comprehensive ETHM regulation across the country [3].

#### 3.4.3. Challenges in ETHM Practice and Practitioners′ Regulation

Regulating traditional herbal medicine (ETHM) methods and practitioners in Ethiopia is still fraught with challenges, despite continuous registration and licensing measures. In addition to issues like illegal street sales and the blending of herbal treatments with prescription medications, many healers continue to operate without proper registration. In general, regulatory efforts have centered more on encouraging legal compliance than on rigorously enforcing the law, though there has been considerable success in urban areas. However, ETHM supervision is often ignored by RHR Agencies, and rural areas remain mostly unsupervised. As a result, most communities that rely on ETHM are no longer fully protected [3].

#### 3.4.4. Scientific Evidence and Validation Challenges

The incorporation of herbal formulations into contemporary healthcare systems is significantly hampered by a lack of strong intellectual property protection and sufficient scientific proof [27]. Although the majority of ETHM practitioners (12 out of 15 in a study) reported no possibility to participate in herbal medicine research, only three people identified opportunities to collaborate with scientific groups. Presently available ETHM products are neither EFDA‐registered nor have been confirmed by authorized labs to satisfy the required efficacy, safety, and quality requirements. Effective regulation is severely hampered by insufficient regulatory frameworks (87.8%) and a dearth of research on herbal medications (90.3%). Because these treatments lack scientific support, conventional medical practitioners become distrustful of them, which further reduces their credibility in modern healthcare settings [4].

#### 3.4.5. Trust and Collaboration Issues Between Stakeholders

One major obstacle to integration is mistrust between researchers and scientific groups and traditional practitioners [4]. Researchers and the scientific community often have concerns about traditional healers, whereas traditional practitioners (80%) say they distrust scientific organizations because they think their information and goods could be stolen. Stakeholder participation is being hampered by the certification of medical products as well as issues with ownership, trust, and patent rights [5]. The absence of committed and accountable ETHM healers′ associations, the lack of agreement and dedication among different ETHM associations, the knowledge gap between healers and conventional medicine professionals, and the incapacity to apply training‐based knowledge are additional challenges to the regulatory environment of traditional medicine. Regulatory authorities face additional challenges due to traditional healers′ reluctance to engage with scientific communities (56.5%). If these trust issues are not addressed, the genuine collaboration needed for a successful integration will continue to elude us [3].

#### 3.4.6. Quality, Safety, and Efficacy Monitoring Challenges

There are many obstacles in ensuring the effectiveness, safety, and quality of traditional medicinal products. The lack of standardized protocols and techniques for gathering, processing, and distributing herbal medicine products was acknowledged by every interviewee. The quality of the raw materials varies since practitioners get medicinal plants from a variety of places, such as their own gardens, marketplaces, rural areas (sometimes 550–650 km from Addis Ababa), and overseas [10]. Although 80% of practitioners gather and prepare medicinal plants themselves, others carry out some tasks. Concerns regarding product safety and uniformity are raised by the variations in harvesting, preparation, and storage methods. Obtaining authentic herbal medications is hampered by adulteration, replacement, and a lack of qualified workers. The obstacles that customs laboratories encounter while analyzing herbal samples range from quality issues to safety and legal considerations [28].

#### 3.4.7. Environmental and Sustainability Challenges

The sustainability of Ethiopia′s medicinal plants is being threatened by environmental deterioration and careless harvesting practices. Communities often collect these plants without proper methods, leading to overharvesting, especially near populated areas, and hastening the demise of valuable species. The issue is made worse by habitat degradation, which reduces the availability of vital pharmacological ingredients needed for traditional medical treatment [14].

The expansion of industry, the disrespect for traditional knowledge, and the lack of agricultural initiatives targeted at the sustainable production of medicinal plants are further dangers. These problems threaten not only the continued existence of traditional medicine but also the possible erasure of untapped pharmacological potential that could contribute to modern medicine through research and conservation efforts [29].

#### 3.4.8. Conclusion for Challenges of the Integration

Ethiopia′s official healthcare system faces numerous challenges in integrating traditional herbal medicine. Although herbal medicines based on African traditional medicine are widely used across the continent for a number of reasons, government healthcare institutions continue to mostly disregard the practice. Among these are the lack of knowledge about the quality, safety, and efficacy of most medicinal plants; stigmatization due to negative attitudes and perceptions; inadequate efforts to preserve indigenous knowledge and medicinal plants; modernization; colonialism′s legacy; exploitation of the communities that possess the knowledge; and the secrecy surrounding traditional medicine practices with few documented adverse reactions [9].

The lack of a reference standard for determining the most effective way to provide patients with traditional treatment is one major barrier. The Ethiopian Food and Drug Authority (FDA) has called on experts and other stakeholders to support national efforts to strengthen and integrate traditional medicines, acknowledging that current efforts are insufficient to create a truly integrated healthcare system that leverages both traditional and modern approaches [30].

### 3.5. Summary Tables of Included Studies and Medicinal Plants

To illustrate the types of evidence available, Table 1 presents selected key studies that informed our analysis. These are representative examples, not an exhaustive list. Table 2 provides examples of Ethiopian medicinal plants with varying levels of research evidence, demonstrating both potential and gaps.

**Table 1:** Summary of key included studies on ETHM integration.

Author (year)	Study type	Region/focus	Main findings related to integration
Dubale et al. (2025)	Cross‐sectional survey	National (regulators and practitioners)	82.3% of regulatory staff lack specific training in traditional herbal medicine (ETHM); highlights critical capacity gaps.
Usure et al. (2024)	Qualitative study	Oromia and SNNPR regions	Identified strong mistrust between traditional healers and researchers; regulatory implementation is fragmented and underresourced.
Bultum et al. (2024)	Data mining/in silico analysis	Not applicable (computational)	Demonstrated a model for validating traditional knowledge using bioinformatics; *Rumex abyssinicus* showed strong wound‐healing potential.
Derso et al. (2024)	Ethnobotanical survey	Jawi District	68% of ethnobotanical knowledge is held by elders, with a 42% loss rate over 15 years due to urbanization.
Tesfaye et al. (2021)	In vitro cytotoxic screening	National (cancer cell lines)	22 Ethiopian medicinal plants exhibited selective cytotoxicity against human cancer cell lines, supporting anticancer drug discovery.
Tuasha et al. (2023)	Systematic review and meta‐analysis	National	Only 40% of the 2019 Traditional Medicine Policy objectives have been met, primarily due to funding and coordination failures.
Kloos (2023)	Review	National	Over two‐thirds of medicinal plants are threatened by overharvesting and habitat loss, underscoring sustainability crises.
WHO (2019)	Global policy report	Global	Outlines integrative, inclusive, and tolerant models for traditional medicine integration into national health systems.
Yikna and Yehualashet (2021)	Review (in vitro/in vivo)	National (antidiabetic plants)	Compiled preclinical evidence on antidiabetic plants, forming a basis for future randomized clinical trials.
Tauchen et al. (2015)	In vitro bioassay	National	18 plant extracts showed significant antioxidant and antiproliferative activities, indicating therapeutic potential.
Getachew et al. (2024)	Cohort study	Eastern Ethiopia	Herbal use during pregnancy was associated with adverse fetal outcomes, emphasizing the need for safety monitoring.
Shiferaw et al. (2020)	Cross‐sectional survey	Addis Ababa (HIV patients)	High prevalence of herbal medicine use among HIV/AIDS patients, highlighting risks of herb‐drug interactions

**Table 2:** Selected Ethiopian medicinal plants and levels of research evidence.

Plant species	Traditional use(s)	Preclinical evidence	Clinical evidence	Safety notes
*Moringa stenopetala*	Diabetes, hypertension	Strong antidiabetic and antihypertensive effects in rodents (Yikna and Yehualashet, 2021)	Limited pilot studies	Generally safe; high doses may cause gastrointestinal upset
*Allium sativum* (Garlic)	Hypertension, infections	Well‐documented antihypertensive and antimicrobial effects	Extensive global data; limited local trials	Safe; may interact with anticoagulants
*Rumex abyssinicus*	Wound healing, skin infections	Wound‐healing activity validated via computational and in vitro models (Bultum et al., 2024)	None reported	Contains oxalates; requires standardization
*Artemisia annua*	Malaria	Strong antiplasmodial activity (global evidence)	Used worldwide; few local RCTs	Contraindicated in pregnancy
*Lobelia giberoa*	Malaria, respiratory ailments	Antiplasmodial and anthelmintic activity in vitro (Debebe et al., 2015)	None reported	Potentially toxic; dose‐dependent safety
*Embelia schimperi*	Intestinal parasites	Anthelmintic efficacy in vivo and in vitro (Debebe et al., 2015)	None reported	Limited safety data
*Withania somnifera*	Stress, inflammation, immunity	Antioxidant, anti‐inflammatory, adaptogenic properties	International studies available; few in Ethiopia	Generally safe; may potentiate sedatives
*Ocimum lamifolium*	Fever, respiratory infections	Antibacterial and antipyretic effects in preclinical studies	None reported	Considered safe in traditional use
*Echinops kebericho*	Febrile illnesses, infections	Antimicrobial and anti‐inflammatory activity reported	None reported	Species under threat; sustainability concern
*Hagenia abyssinica*	Tapeworm infection	Established anthelmintic efficacy	Some historical clinical use	Emetic at high doses; precise dosing required
*Aloe spp.*	Skin conditions, wounds, laxative	Wound healing and anti‐inflammatory effects documented	Some clinical studies on wound healing	Laxative effect may cause electrolyte imbalance
*Zingiber officinale* (Ginger)	Nausea, inflammation, digestion	Strong antiemetic and anti‐inflammatory evidence	Extensive global clinical data	Safe; may interact with blood thinners
*Trigonella foenum-graecum* (Fenugreek)	Diabetes, galactagogue	Hypoglycemic and lipid‐lowering effects in animals	Clinical trials for diabetes globally	May significantly lower blood glucose
*Vernonia amygdalina*	Malaria, diabetes, gastrointestinal issues	Antiplasmodial, antidiabetic, and antimicrobial activities	Limited clinical studies	Bitter taste; considered safe at medicinal doses
*Croton macrostachyus*	Malaria, wounds, digestive disorders	Antiplasmodial, antimicrobial, and anti‐inflammatory activities	None reported	Toxic in high doses; preparation critical

## 4. Discussion

### 4.1. Successful Integration Models From Other Countries

Three types of traditional medicine use are distinguished by the WHO: integrative, inclusive, and tolerant. In integrative systems such as those in China, the Republic of Korea, and Vietnam, traditional medicine is fully integrated into all aspects of healthcare delivery, including policy, education, and clinical practice, and is legally recognized.

Although they may not fully incorporate it into the healthcare system, nations with inclusive systems such as the United Kingdom, Germany, Canada, Australia, Ghana, Nigeria, Mali, and Equatorial Guinea recognize traditional medicine as a legitimate medical practice. Because they are heavily influenced by Western medicine, some traditional methods are only partially legal in tolerant systems, such as the one in New Zealand. China′s integration model, which is noteworthy for its thorough integration of traditional medicine with modern healthcare, exemplifies a highly institutionalized and policy‐backed approach. China′s experience offers insights into institutional approaches, though adaptation to Ethiopia′s context would be necessary [31].

### 4.2. China′s Model of Integration

Beginning in the late 1950s, traditional Chinese medicine (TCM) was included into China′s national healthcare system [32]. TCM is widely accepted and used in clinical practice, research, teaching, and other facets of healthcare [33]. With many TCM medicines going through extensive testing like traditional pharmaceuticals, the Chinese approach places a strong emphasis on the scientific validation of traditional therapies. Through a more modern and transparent research and development approach, it is projected that TCM will integrate with modern medicine by 2035 [34]. When proper legislation, research, and educational frameworks are in place, the Chinese model shows how traditional and contemporary medicine can coexist successfully [33].

### 4.3. India′s Approach to Traditional Medicine Integration

A thorough framework for incorporating Ayurvedic medicine with contemporary healthcare has been built in India. As per the Indian government′s plan, AYUSH (Ayurveda, Yoga, Naturopathy, Unani, Siddha, and Homeopathy) services are currently located in primary health centers (PHCs) and community health centers (CHCs). This colocation strategy allows resources and infrastructure to be shared while maintaining the distinctive features of traditional systems. Since 2000, the process of integrating Ayurveda with modern medicine in the Indian healthcare system has changed drastically due to significant investments in research, education, and clinical applications [33].

### 4.4. Ghana′s Experience in Traditional Medicine Integration

Ghana has made notable progress in integrating traditional medicine into its healthcare system. In 2012, the government approved the integration of traditional medicine into the healthcare system of Ghana [7]. A number of rural hospitals in the nation have already incorporated traditional medicine, opening the door for hybrid healthcare approaches. The Ghana FDA and the Traditional Medicine Practice Council (ETHMPC) are responsible for registering, licensing, and regulating the activities of practitioners of traditional medicine in Ghana [31]. The legitimacy and caliber of traditional medicine in the nation are enhanced by the Traditional Medicine Unit of Kwame Nkrumah University of Science and Technology (KNUST) Faculty of Pharmacy, which trains specialists in its practice [5].

### 4.5. Current Ethiopian Herbal Medicine Researches In Vitro or Clinical Trial

#### 4.5.1. Current Ethiopian Herbal Medicine Researches In Vitro and In Vivo

Recent studies in Ethiopia have extensively evaluated medicinal plants using in vitro methods for various therapeutic activities:

##### 4.5.1.1. Antidiabetic Potential

Research conducted at Debre Berhan University of both in vitro and in vivo has demonstrated the strong antidiabetic effects of several Ethiopian medicinal herbs [6]. The findings support conventional applications and offer a strong scientific foundation for future clinical trials in the treatment of diabetes.

##### 4.5.1.2. Antioxidant and Antiproliferative Effects

Research on 18 Ethiopian therapeutic plants has shown significant antiproliferative and antioxidant properties [7]. These results show significant therapeutic potential and are especially pertinent to the treatment of cancer and disorders linked to oxidative stress.

##### 4.5.1.3. Antiparasitic and Antiplasmodial Activities

Effectiveness against parasites like *Haemonchus contortus* has been demonstrated by in vitro anthelmintic tests conducted on seeds from traditional medicinal herbs. Some plants, like *Lobelia giberoa*, which has long been used to treat malaria, have shown some degree of antiplasmodial activity [8]. A number of plants have also been shown to exhibit antileishmanial activity, suggesting that they may play a part in the management of leishmaniasis.

##### 4.5.1.4. Antibacterial and Anticancer Investigations

To find new antimicrobial agents, Ethiopian medicinal plants that are well‐known for their therapeutic uses in traditional medicine have had their antibacterial activity assessed in vitro [9]. Studies on the cytotoxic and antiproliferative qualities of these plants also indicate that they could be used for the manufacture of anticancer medications in the future [11].

##### 4.5.1.5. Current Clinical Trials in Ethiopia

Even while preclinical in vitro and in vivo studies on Ethiopian medicinal plants have created a solid foundation for clinical research, comprehensive clinical trials are still rare [35]. This discrepancy is mostly due to the absence of exact, standardized methods created especially for clinical studies in Ethiopian traditional medicine [3]. The majority of current clinical research focuses on evaluating safety, consumption patterns, and potential adverse effects, particularly in relation to vulnerable populations such as pregnant women, those with chronic illnesses, and those living with HIV/AIDS [36–38].

Regulatory frameworks are currently being developed to improve clinical trial oversight and streamline the incorporation of traditional medicine into Ethiopia′s official healthcare system [3]. However, recent research consistently emphasizes the urgent need for better designed clinical trials to validate the safety and therapeutic effectiveness of herbal medicines that have long been used to treat cancer, diabetes, and infectious diseases [4]. Strong clinical research and regulation are necessary to enhance the evidence‐based integration of traditional treatments into contemporary medical practice [39].

## 5. Limitations of This Review

It is a comprehensive synthesis of contemporary literature on the integration of ETHM, but it has several limitations. First, the exclusion of non‐English language publications may introduce a language bias, potentially omitting relevant studies published in local Ethiopian or regional languages. Second, while major international and regional databases were searched, some grey literature or locally published reports may have been overlooked, compromising the policy analysis′ completeness. Moreover, as the reviews were comprehensive, the search was not systematic and may have missed some relevant studies. Third, the studies included have very different designs, ranging from qualitative interviews and cross‐sectional surveys to in vitro experiments and policy analyses. This heterogeneity precludes quantitative synthesis (meta‐analysis), necessitating a qualitative, interpretive approach to evidence synthesis. These limitations should be taken into account when interpreting the findings and making broad recommendations.

## 6. Linking Evidence to Action: A Framework for Integration

This review′s evidence synthesis reveals that integration challenges are interdependent, necessitating a coordinated, strategic response. Direct mapping of key evidence gaps to actionable policy and research initiatives is critical for progress. For instance, the critical scarcity of clinical trials fewer than five since 2015 requires the formation of a national traditional medicine research consortium as well as the establishment of dedicated funding streams to prioritize and conduct Phase II/III trials on the most promising medicinal plants.

Empirical findings of heavy metal contamination and adulteration in market samples [8] highlight the urgent need to establish a national quality surveillance and pharmacovigilance program for herbal products that includes routine screening and adverse event reporting.

The identified lack of a specialized regulatory unit and standardized training for regulators [3, 4] leads to the recommendation of establishing a dedicated THM directorate within the EFDA and developing accredited certification programs.

Evidence of rapid loss of indigenous knowledge and medicinal plant species [10, 12, 13] supports the urgent need for digital knowledge preservation (EthHerb) and community‐based conservation and cultivation programs.

This evidence‐to‐action framework ensures that the proposed recommendations are not abstract, but rather direct and logical responses to the documented barriers in the Ethiopian context. This targeted approach allows stakeholders to efficiently allocate resources and track progress against specific, evidence‐based gaps.

## 7. Future Research and Innovation

Future work should concentrate on calibrated, quantitative pipelines for ethnobotanical research. Combining systematic field data with computational models, inspired by cross‐disciplinary approaches to quantitative profiling in materials science, could transform qualitative traditional knowledge into reproducible spatial maps of plant use, efficacy, and conservation priority [40, 41].

## 8. Conclusion

The integration of ETHM with modern pharmacological systems has the potential to strengthen national healthcare delivery, preserve cultural heritage, and may contribute to global knowledge on medicinal plants. Although Ethiopia′s rich ethnobotanical knowledge and high public reliance on traditional medicine provide a strong foundation, this review has systematically identified that realizing this potential is hindered by interconnected challenges in regulation, scientific validation, quality control, stakeholder collaboration, and sustainability.

A critical synthesis of the findings reveals that these challenges cannot be addressed in isolation. Therefore, effective integration requires a structured, multiphase framework that aligns policy reform, capacity building, and research innovation. This framework must respect and build upon the existing community‐based knowledge systems of traditional healers while introducing necessary safeguards and standards for safety and efficacy.

The proposed shift from a fragmented “EGO system” to a collaborative “ECO system” is not merely rhetorical; it necessitates concrete, actionable strategies. These include establishing a specialized regulatory unit within the EFDA, launching targeted interdisciplinary research programs, and implementing digital tools for knowledge preservation and supply chain monitoring. By adopting a phased implementation roadmap with clear short‐, medium‐, and long‐term goals, Ethiopia can help narrow the gap between traditional and modern medicine. Such an approach would not only fulfill the unmet healthcare needs of its population but could also help Ethiopia develop a more integrated healthcare model.

## 9. Recommendation

Based on the synthesis of evidence in this review, the following phased and evidence‐based recommendations are proposed to facilitate the effective and sustainable integration of ETHM into the national healthcare system.

### 9.1. Short‐Term Priorities: Foundational and Regulatory Strengthening

✓ Establish a dedicated ETHM unit: create a specialized traditional herbal medicine directorate or unit within the EFDA with a clear mandate, budget, and trained personnel.
✓ Launch a national digital repository: develop “EthHerb,” a centralized digital database to document medicinal plants, their uses, preparation methods, and associated traditional knowledge, involving healers in the process with clear protocols for intellectual property protection.
✓ Initiate priority safety studies: conduct immediate toxicological screening and heavy metal/adulterant analysis for the top 20 most widely traded herbal products in urban markets like Addis Ababa.

### 9.2. Medium‐Term Strategies: Capacity Building and Research Integration

➢ Implement a dual‐track training system:
  ✓ For practitioners: develop certificate programs in basic pharmacology, hygiene, and record‐keeping for traditional healers, complementing not replacing their apprenticeship training.
  ✓ For regulators and health professionals: integrate modules on ETHM into the curricula of pharmacy, medical, and public health schools to foster mutual respect and interdisciplinary understanding.
➢ Foster collaborative research consortia: fund and establish formal partnerships between universities (e.g., Addis Ababa University and Debre Berhan University), the EFDA, and healer associations to design and conduct clinical trials on priority plants (e.g., for diabetes, malaria, and wound healing).
➢ Pilot and integrated care model: select three to five primary healthcare centers in diverse regions to pilot the colocation of a certified traditional practitioner alongside modern health workers, with shared patient records and referral protocols.

### 9.3. Long‐Term Goals: Systemic Integration and Sustainability

✓ Enact a comprehensive traditional medicine act: develop and pass standalone legislation that provides a clear legal identity for ETHM, defines practices and products, and establishes robust intellectual property and benefit‐sharing mechanisms.
✓ Develop and scale sustainable cultivation programs: transition key medicinal plants from wild harvesting to controlled cultivation by establishing community‐based medicinal plant gardens and providing agrotechnical support to ensure consistent quality and species conservation.
✓ Achieve full health system integration: incorporate validated and quality‐assured traditional treatments into the Essential Medicines List and national health insurance scheme, ensuring affordability and formal access.
❖ By adopting this staged, evidence‐informed framework, Ethiopia can move toward a more integrated healthcare system that respects traditional knowledge while addressing safety concerns.

**Nomenclature**
AJOL: African Journals Online
COVID‐19: Corona Virus Disease‐2019
ECO: Embracing Collective Outcomes
EFDA: Ethiopian Food and Drug Authorities
EGO: Edging God Out (or) Everything Good Only‐for‐me
ETHM: Ethiopian traditional herbal medicine
ETHM: Traditional herbal medicine
ETHM: Traditional medicine
MM: Modern medicine
RHR: Regional Health Regulatory
WHO: World Health Organization

## Author Contributions

K.D.A., B.A., B.M., and A.S.Y. have designed the method, conducted the review, and wrote the original draft. B.M. and K.D.A. have supervised the review.

## Funding

No funding was received for this manuscript.

## Disclosure

All authors have approved the final version.

## Ethics Statement

The authors have nothing to report.

## Consent

The authors have nothing to report.

## Conflicts of Interest

The authors declare no conflicts of interest.

## Data Availability

The data that support the findings of this study are available on request from the corresponding author. The data are not publicly available due to privacy or ethical restrictions.
